# SOCIAL DETERMINANTS OF DEPRESSION AMONG OLDER ADULTS IN THE UAB STUDY OF AGING: THE ROLE OF RACE-BASED DISCRIMINATION

**DOI:** 10.1093/geroni/igad104.3594

**Published:** 2023-12-21

**Authors:** Katie Buys, David Buys, Pamela Bowen, Richard Kennedy

**Affiliations:** University of Alabama at Birmingham, Birmingham, Alabama, United States; Mississippi State University, Mississippi State, Mississippi, United States; University of Alabama at Birmingham, Birmingham, Alabama, United States; University of Alabama at Birmingham, Birmingham, Alabama, United States

## Abstract

Mental health is known to be influenced by social determinants of health (SDoH). However, little is known about how racial discrimination, in the context of other social factors, impacts the mental health of older adults. The focus of this research is specifically on the different effects of racial discrimination in that broader context on depression diagnosis vs. symptomology. Using the UAB Study of Aging’s cohort of 1000 Alabamians 65+ balanced on rural/ urban status, sex (men/women), and race (Black/white), we evaluate the impact of SDoH, including race-based discrimination, on clinically verified depression diagnosis and on depressive symptomology using the Geriatric Depression Scale. Other independent variables include environmental factors (eg: neighborhood disadvantage), status and assets, interpersonal factors, and fixed demographic characteristics. Using generalized estimating equations, we found that experience of racial discrimination is associated with a depression diagnosis (OR: 2.33) independent of race and other factors. Other variables that were significant predictors of having a verified depression diagnosis include life-space mobility and race. Factors associated with depressive symptomology include fear of crime, financial difficulty, social support, life-space mobility, race (Black), and sex (female). While demonstrating that experience of race-based discrimination was associated with depression diagnosis is not surprising, finding in an otherwise identical model, that it was not associated with symptomology was unexpected. Authors will discuss some potential reasons for this counterintuitive outcome and future directions for better understanding this inconsistency.

